# Supplementary material for: Nuclear oligo hashing improves differential analysis of single-cell RNA-seq
Source: Nat Commun. 2022 May 13;13:2666. doi: 10.1038/s41467-022-30309-4 (PMC9106741; doi:10.1038/s41467-022-30309-4)
Supplement: Supplementary file 1 — Supplementary Information [file 41467_2022_30309_MOESM1_ESM.pdf]

Supplemental Information

Nuclear oligo hashing improves differential analysis of single-cell RNA-seq

Hyeon-Jin Kim, Greg Booth, Lauren Saunders, Sanjay Srivatsan, José L. McFaline-Figueroa, Cole Trapnell

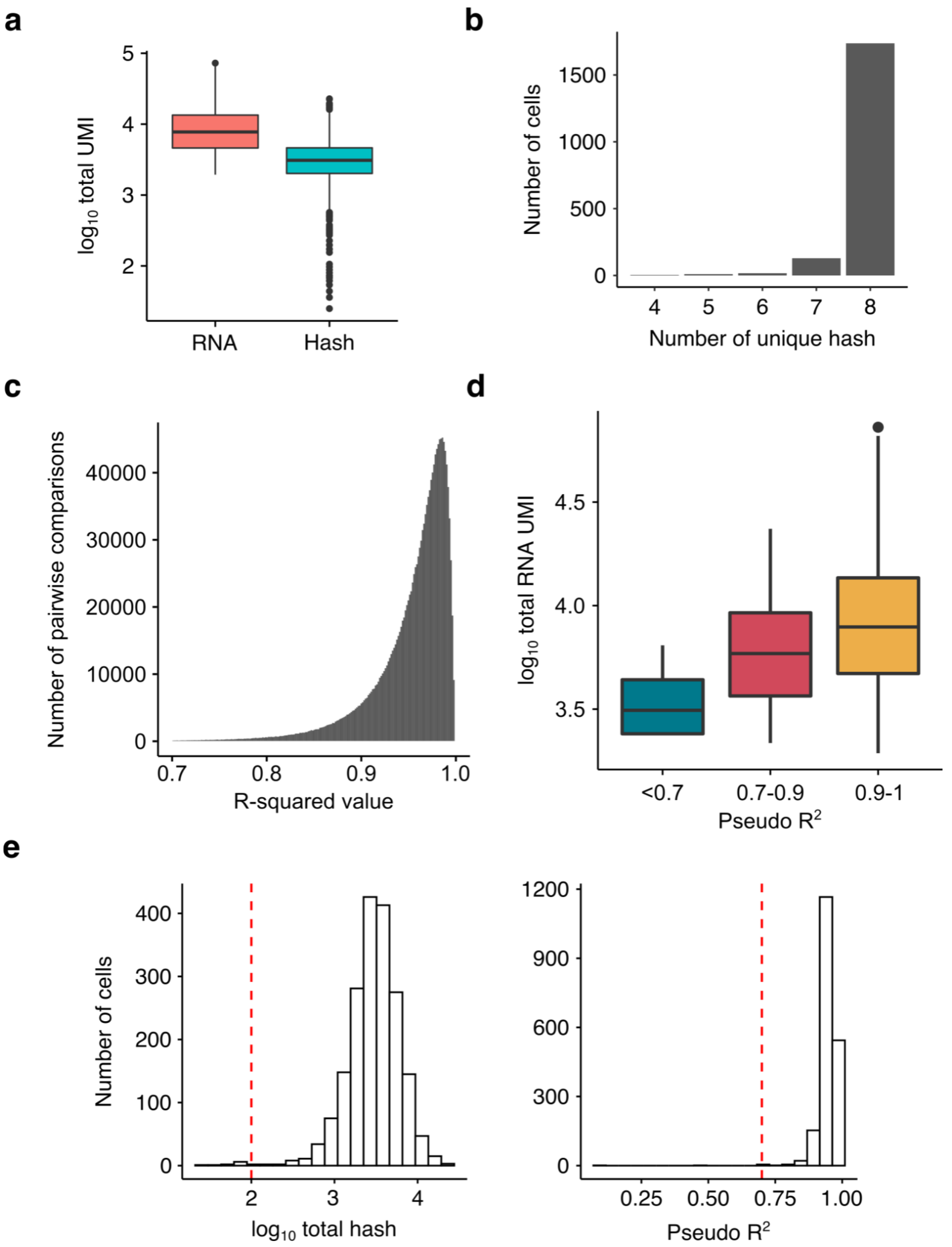

Supplementary Fig. 1: Hash ladder is captured within nuclei in sci-RNA-seq experiments. (**a**) Boxplot of total RNA and hash UMI count per cell (n = 1897 cells). (**b**) Barplot showing the number of cells with a number of unique hash molecules with read count greater than 1. (**c**) Histogram showing correlations in observed hash ladder UMIs between all pairs of cells. (**d**) Boxplot showing the distribution of total RNA UMIs for cells binned according to their pseudo R-squared values of the fitted hash ladder calibration curves (n = 1897 cells). (**e**) Quality filter that was used to filter out low quality cells. Red dashed line shows total hash count (left, > 100) and pseudo R-squared value (right > 0.7) cutoff. The centerline of the boxplots in **a,d** indicates the median, the box displays the first and third quartile, and the whiskers show the 1.5 interquartile range (IQR). Outlier values are displayed as points.

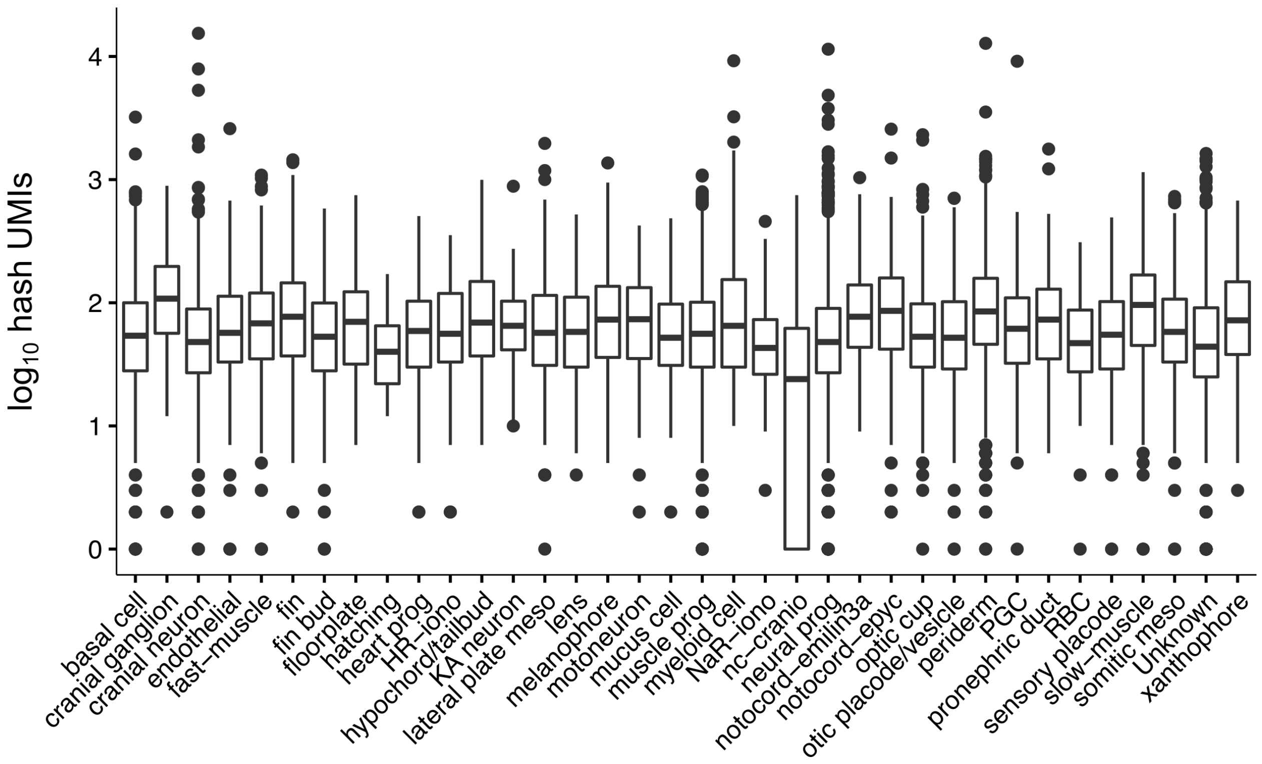

Supplementary Fig. 2: **Hashing is compatible with whole zebrafish.** Boxplot showing hash UMI counts for each cell type identified in zebrafish embryos using known gene markers (n = 29,800 cells). Briefly, single cell suspensions were collected from zebrafish embryos by dissociating in trypLE at 37C. The cells were then lysed and fixed with 50 picomoles of single hash species as described in Methods. The centerline of the boxplots indicates the median, the box displays the first and third quartile, and the whiskers show the 1.5 IQR. Outlier values are displayed as points.

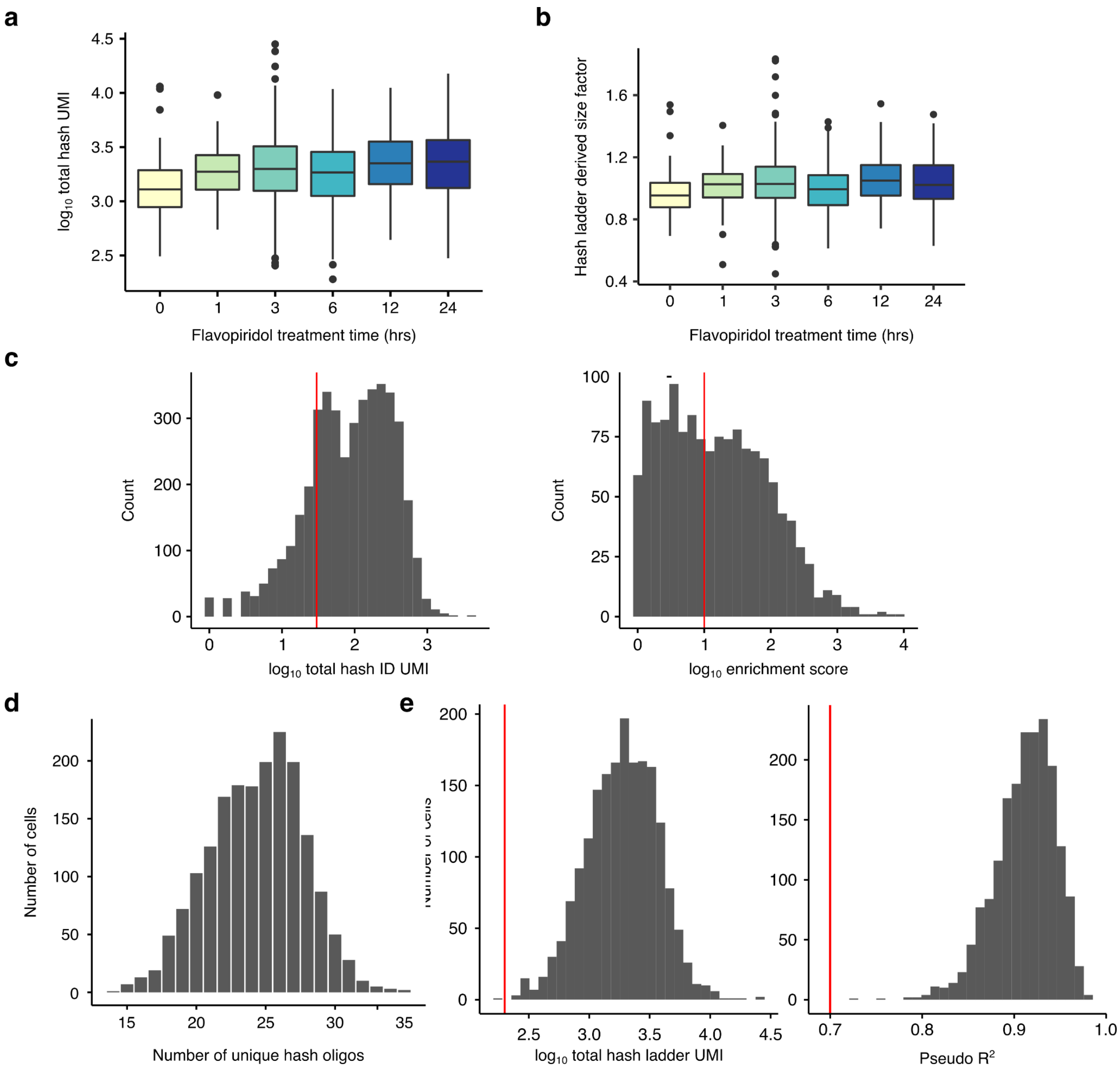

Supplementary Fig. 3: **Quality control of cells from the flavopiridol time course experiment.** Boxplots showing the hash ladder counts per cell (**a**) and hash ladder derived size factor values (**b**) for each flavopiridol treatment time (n = 1370 cells). The centerline of the boxplots indicates the median, the box displays the first and third quartile, and the whiskers show the 1.5 IQR. Outlier values are displayed as points. (**c**) Histogram showing the distribution of the number of ID hash UMIs detected per cell (left) and top hash ID enrichment score of most abundant hash vs. second most abundant hash (right). Cells with lower than 30 ID hash UMIs and enrichment score below 10 (red lines) were filtered out. (**d**) Barplot showing the number of cells with a number of unique hash ladder molecules with read count greater than 1. (**e**) Histograms showing distribution of total hash ladder UMIs per cell (left) and pseudo R-squared value of the hash ladder calibration line (right). Red dashed line shows total hash count (left, > 100) and pseudo R-squared value (right > 0.7) cutoff.

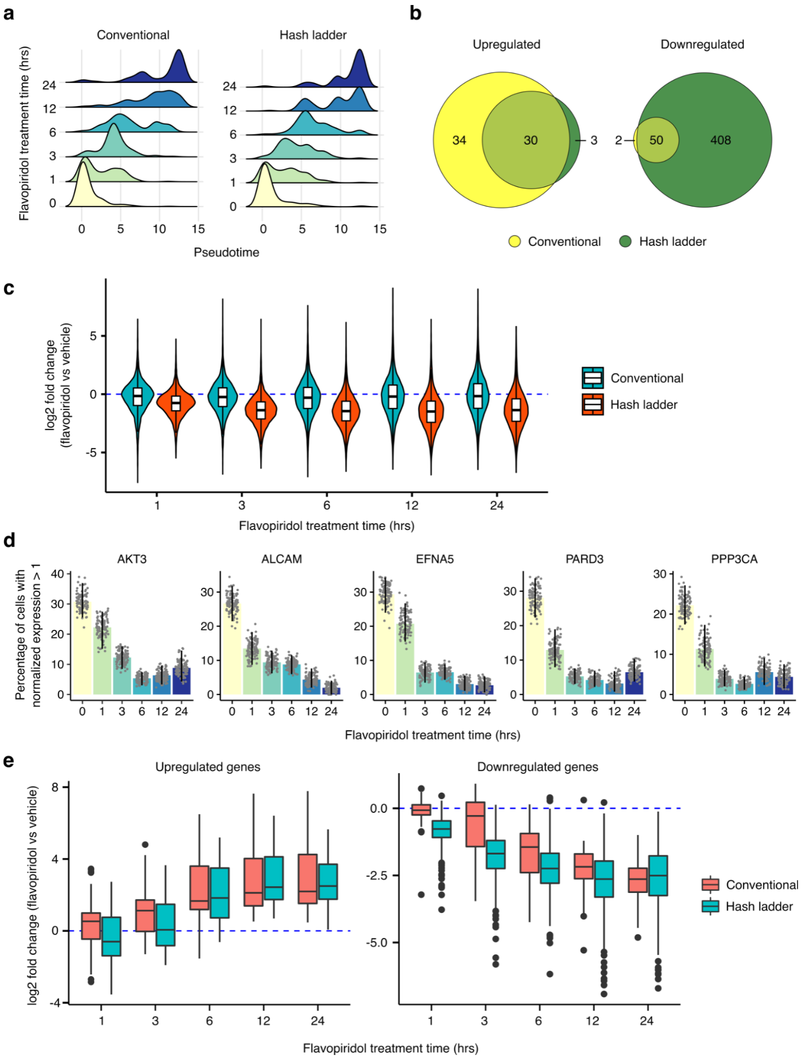

Supplementary Fig. 4: **Hash ladder-based normalization enhances the detection of global reduction in transcription caused by flavopiridol.** (**a**) Pseudotime distribution of cells at each treatment time using conventional and hash ladder normalized expression values. (**b**) Venn diagrams showing the overlap of differentially upregulated and downregulated genes along the flavopiridol pseudotime trajectory (log2 fold change > 1) between the conventional and hash ladder normalization methods. (**c**) Violin plot comparing the log2 fold changes of average conventional and hash ladder normalized expression values of all expressed genes (vehicle vs. flavopiridol treated; n = 11,637 genes). (**d**) Hash ladder normalized expression levels of genes involved in cell migration and neurogenesis at different flavopiridol treatment times. Bars represent the percentage of cells with normalized expression value greater than 1, and the error bars show the 95% confidence interval obtained using a bootstrap method (n = 100 bootstrap samples). (**e**) Boxplot comparing the log2 fold changes of average conventional (n = 61, 59 genes) and hash ladder (n = 27, 407 genes) normalized expression values of upregulated and downregulated genes (vehicle vs. flavopiridol treated). The centerline of the boxplots in **c**,**e** indicates the median, the box displays the first and third quartile, and the whiskers show the 1.5 IQR. Outlier values are displayed as points.

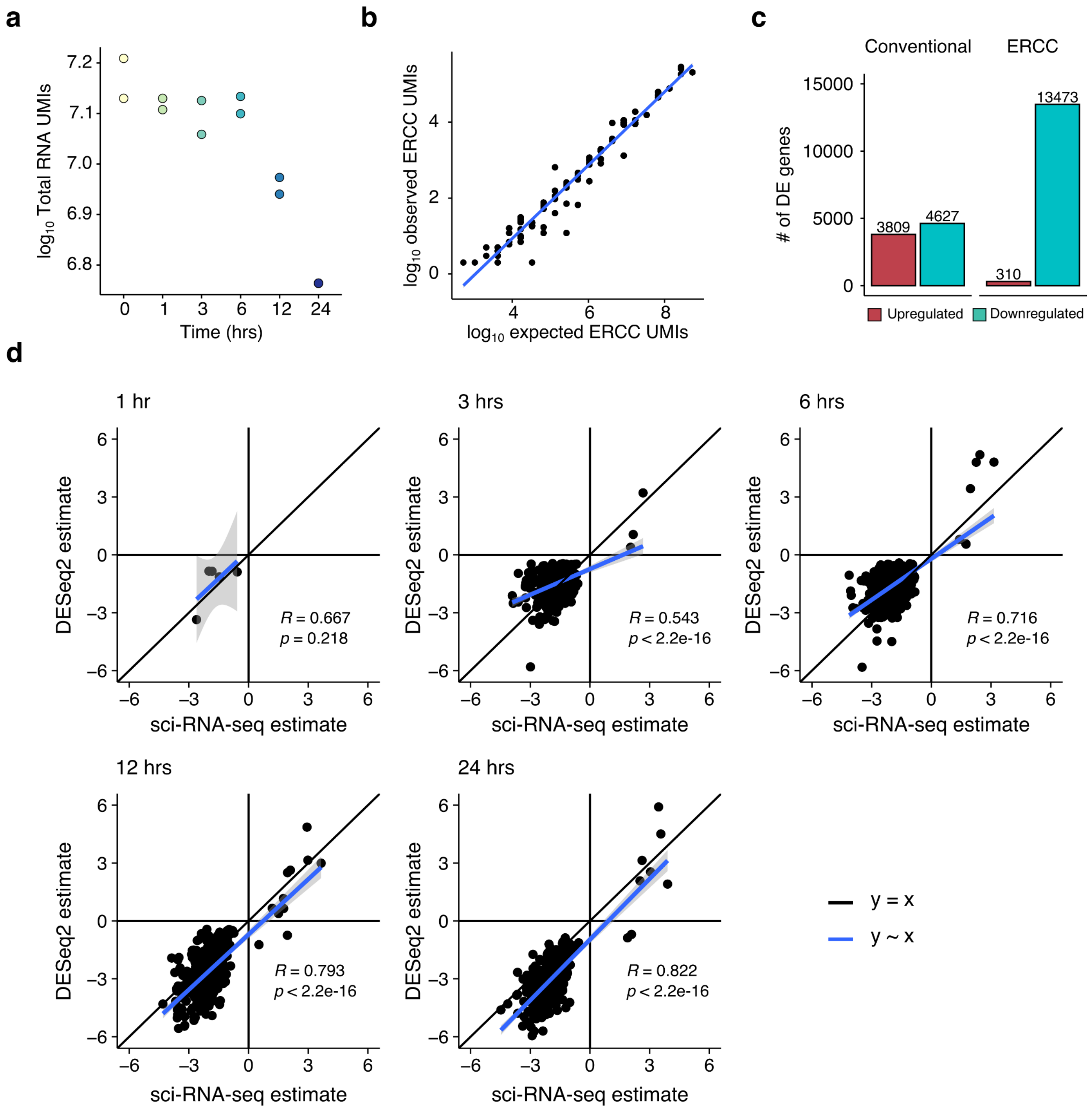

Supplementary Fig. 5: Bulk RNA-seq corroborates scRNA measurements in flavopiridol timecourse experiment. (**a**) Log10 total RNA UMIs for cells treated with flavopiridol at each timepoint, measured by bulk RNA-seq. n = 2 replicates. (**b**) Correlation of expected and observed ERCC spike-in molecules for vehicle cells. (**c**) Number of differentially expressed genes found using conventional and ERCC normalization (vehicle vs 24 hours). (**d**) Scatterplots of statistically significant gene estimates derived from sci-RNA-seq data using Monocle3 treating time as a discrete variable and estimates derived from the bulk RNA-seq data using DESeq2. Black line indicates y=x and the blue line indicates the fit.

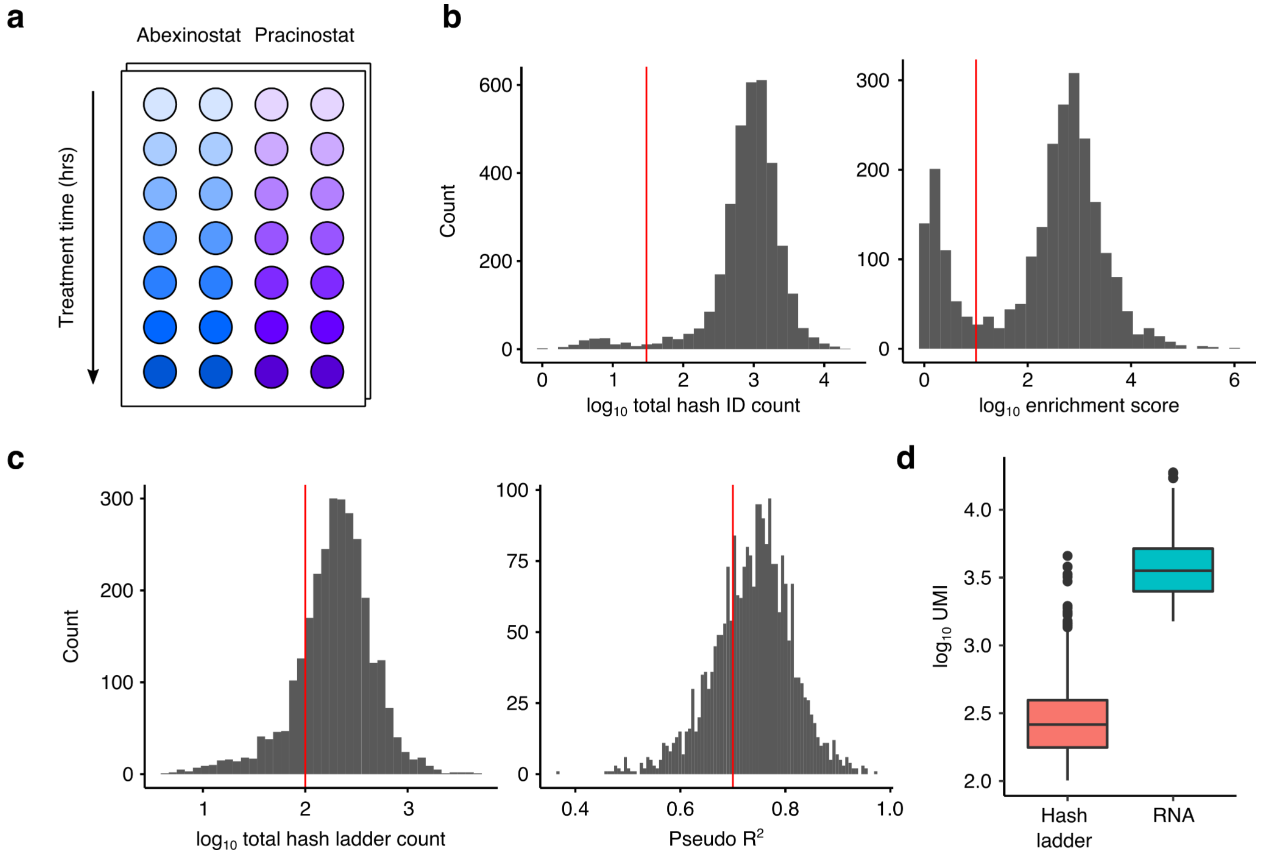

Supplementary Fig. 6: **Quality control of cells from the histone deacetylase (HDAC) inhibitor time course experiment.** (**a**) Experimental design of the HDAC inhibitor time course experiment. (**b**) Histogram showing the distribution of the number of ID hash UMIs detected per cell (left) and top hash ID enrichment score of most abundant hash vs. second most abundant hash (right). Cells with lower than 30 ID hash UMIs and enrichment score below 10 (red lines) were filtered out. (**c**) Histograms showing distribution of total hash ladder UMIs per cell (left) and pseudo R-squared value of the hash ladder calibration line (right). Red dashed line shows total hash count (left, > 100) and pseudo R-squared value (right > 0.7) cutoff. (**d**) Boxplot showing the distribution of the number of UMIs per cell (n = 1548 cells over two replicates) recovered for the hash ladder (median 261 UMIs) and the transcriptome (median 3552 UMIs). The centerline of the boxplots indicates the median, the box displays the first and third quartile, and the whiskers show the 1.5 IQR. Outlier values are displayed as points.

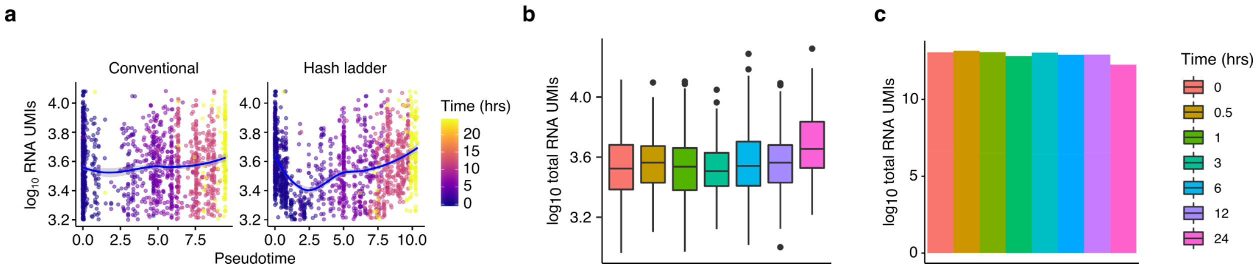

Supplementary Fig. 7: Transient reduction of total RNA UMIs along the HDACi trajectory is only visible in the pseudotime space. (**a**) Scatterplot of total RNA UMIs as a function of inferred pseudotime values. (**b**) Boxplot showing the distribution of total RNA UMIs of cells grouped by HDACi treatment time (n = 1548 cells over two replicates). The centerline of the boxplots indicates the median, the box displays the first and third quartile, and the whiskers show the 1.5 IQR. Outlier values are displayed as points. (**c**) Barplot of total RNA UMIs measured by bulk RNA-seq. Colors indicate HDACi treatment time.

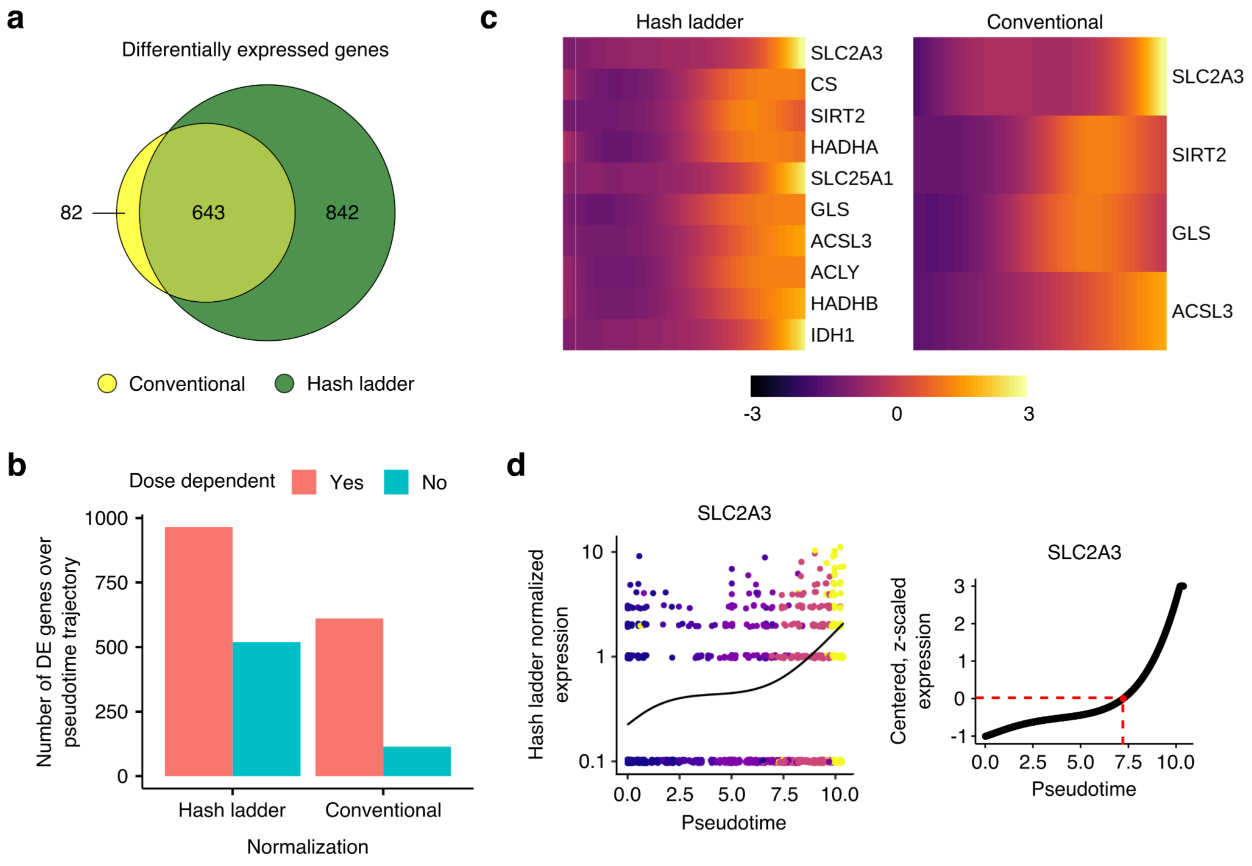

Supplementary Fig. 8: **Hash ladder-based normalization recovers a higher number of previously identified differentially expressed (DE) genes along the HDAC inhibitor time course trajectory.** (**a**) Venn diagrams showing the overlap of differentially expressed genes along the HDAC inhibitor pseudotime trajectory between the conventional and hash ladder normalization methods. (**b**) Barplots showing the number of pseudotime dependent differentially expressed genes that are also found to be pseudodose dependent in the sci-Plex experiment performed by Srivatsan et al. *Science* 2020. (**c**) Heatmaps showing row-centered and z-scaled expression kinetics of DE genes involved in acetyl-CoA synthesis using the hash ladder and conventional normalization approaches. (**d**) Demonstration of how the onset of cellular metabolic crisis is estimated. First, hash ladder normalized expression values were fitted using the smoothed HDAC inhibitor pseudotime values via a natural spline function (left). The fitted expression values were then centered and z-scaled and plotted against the pseudotime values to identify the time at which the centered, z-scaled value is equal to zero (right).

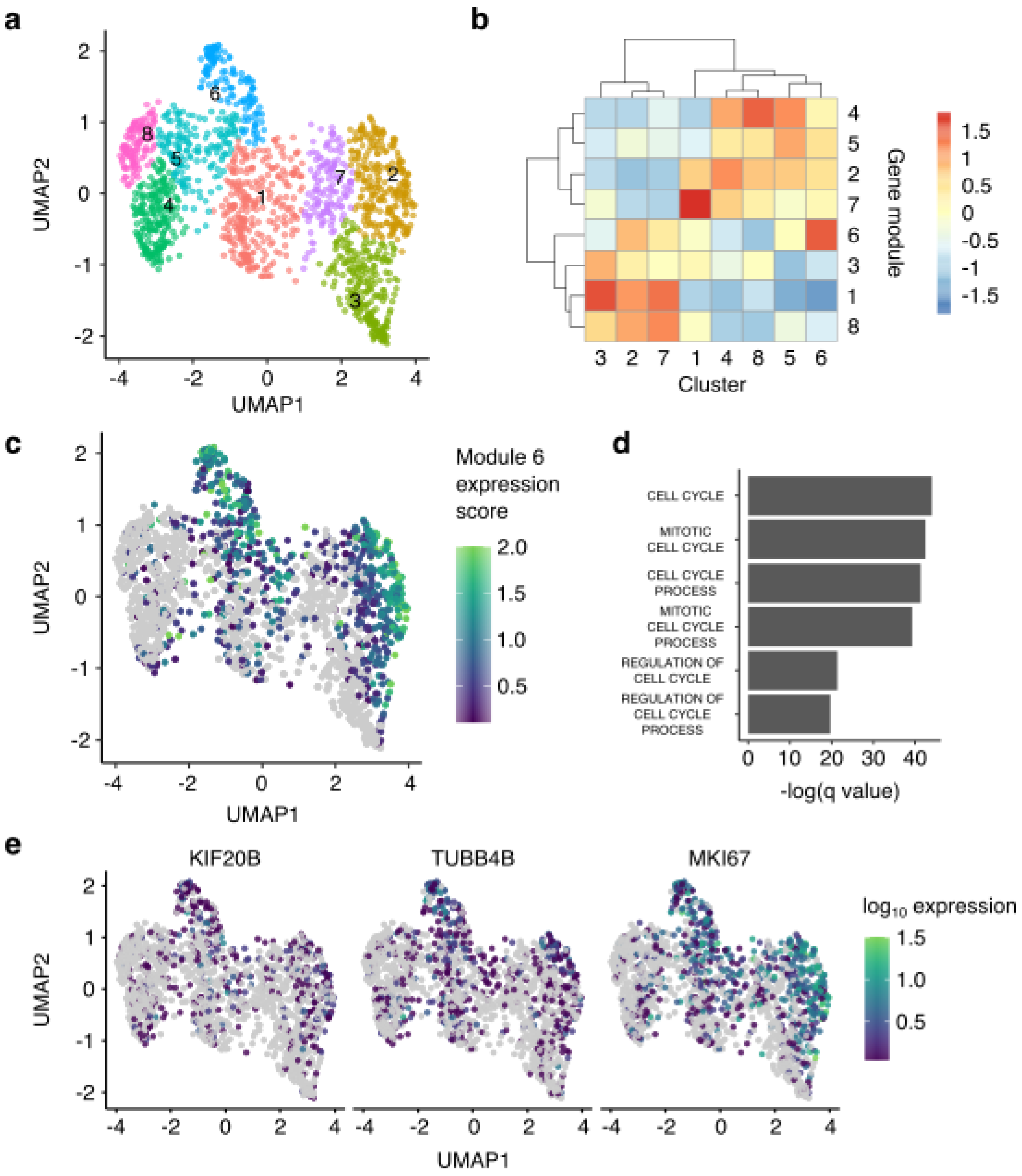

Supplementary Fig. 9: **Hash ladder-based normalization recovers an additional branch in the UMAP space.** (**a**) UMAP projection of HDAC inhibitor treated cells, colored by cluster identity from Louvain clustering. (**b**) Heatmap showing centered and z-scaled aggregated expression of genes that are co-regulated ("modules") across the clusters. Cluster 6 is enriched for genes in module 6. (**c**) UMAP projection of HDAC inhibitor treated cells, colored by expression score for module 6. (**d**) Barplot showing the result of gene set enrichment analysis of module 6 genes using the Gene Ontology biological processes gene sets (FDR < 1e-19). (**e**) UMAP projection of HDAC inhibitor treated cells, colored by genes involved in cell cycle processes.

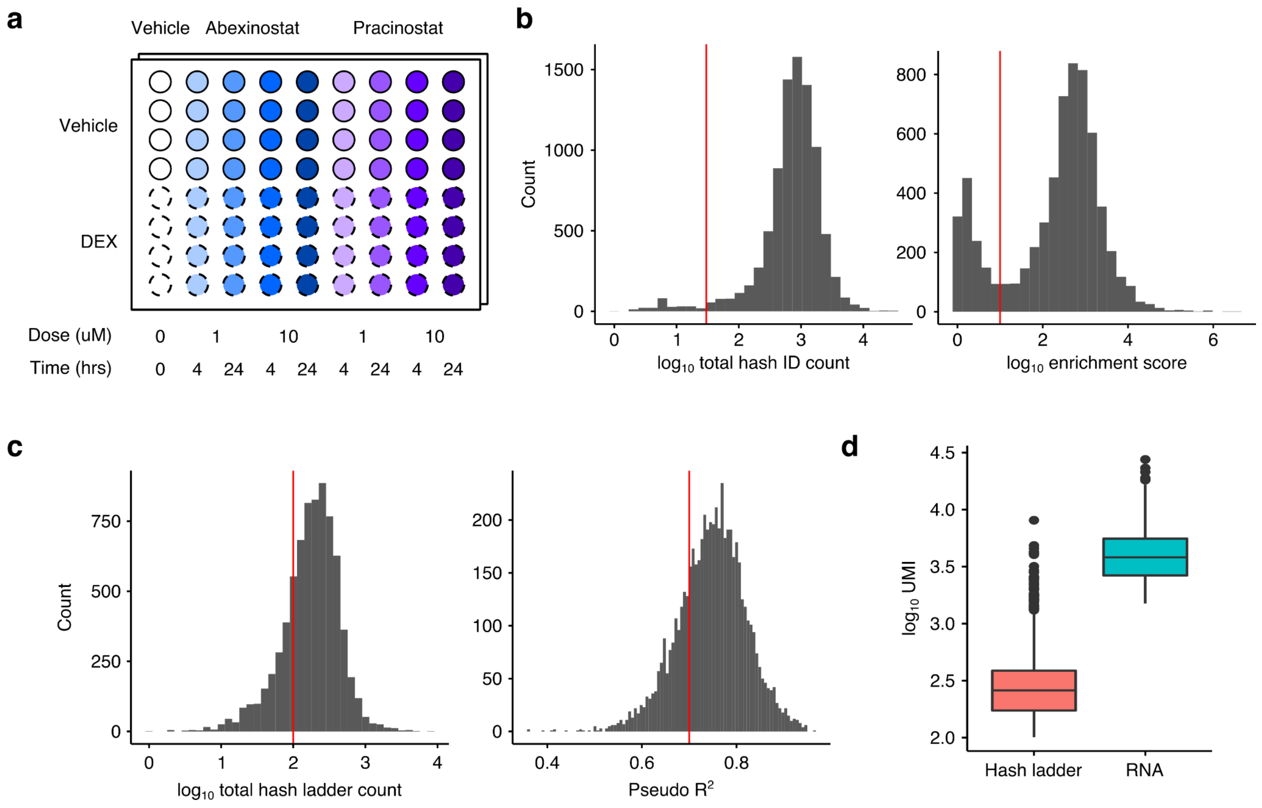

Supplementary Fig. 10: **Quality control of cells from the histone deacetylase (HDAC) inhibitor and dexamethasone (DEX) co-treatment experiment.** (**a**) Experimental design of the HDAC inhibitor and DEX treatment experiment. The dose and treatment time of HDAC inhibitors prior to DEX treatment are shown below. (**b**) Histogram showing the distribution of the number of ID hash UMIs detected per cell (left) and top hash ID enrichment score of most abundant hash vs. second most abundant hash (right). Cells with lower than 30 ID hash UMIs and enrichment score below 10 (red lines) were filtered out. (**c**) Histograms showing distribution of total hash ladder UMIs per cell (left) and pseudo R-squared value of the hash ladder calibration line (right). Red dashed line shows total hash count (left, > 100) and pseudo R-squared value (right, > 0.7) cutoff. (**d**) Boxplot showing the distribution of the number of UMIs per cell (n = 3710 cells over two replicates) recovered for the hash ladder (median 260 UMIs) and the transcriptome (median 3816 UMIs). The centerline of the boxplots indicates the median, the box displays the first and third quartile, and the whiskers show the 1.5 IQR. Outlier values are displayed as points.

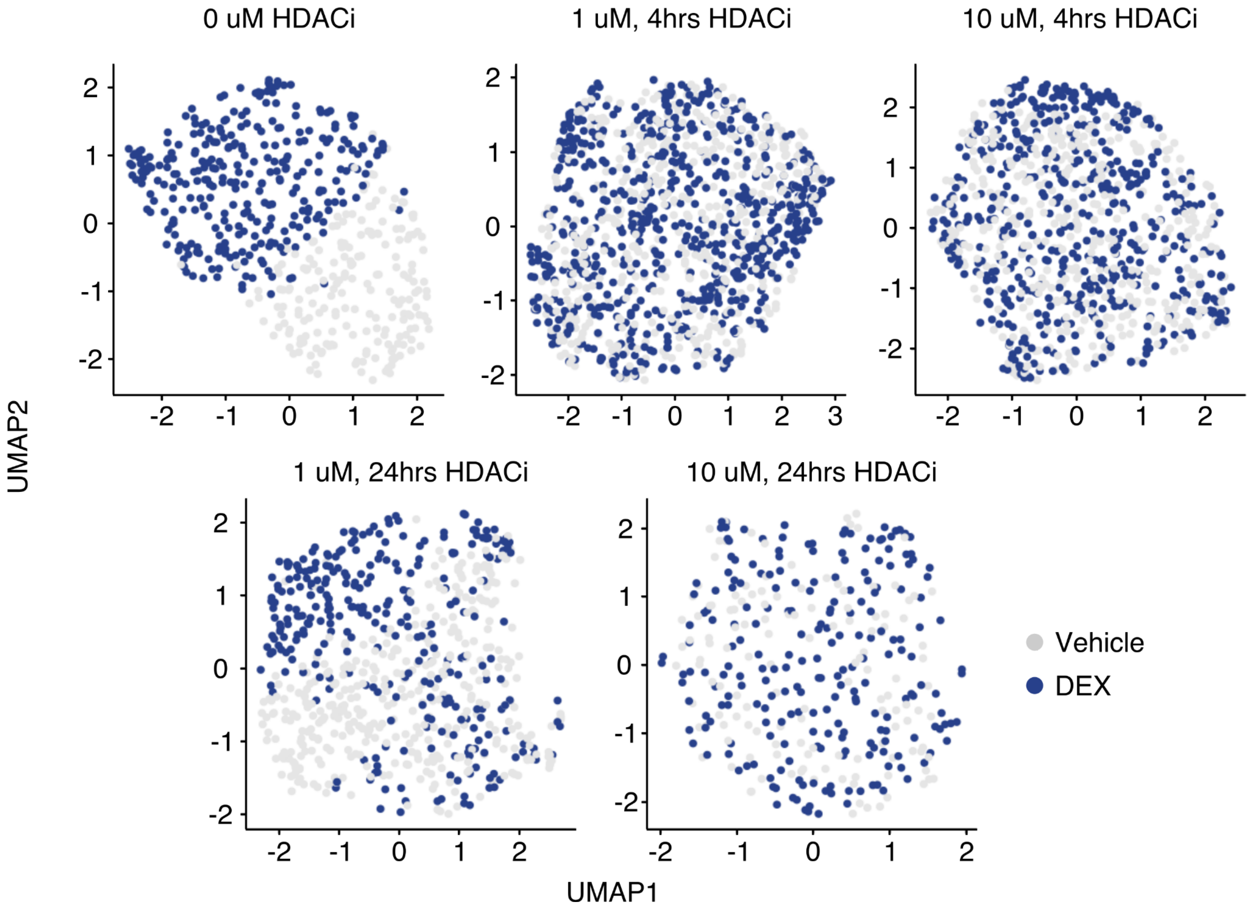

Supplementary Fig. 11: UMAP embeddings of HDACi and DEX treated cells. UMAP embeddings from Fig. 4b were faceted by HDACI dose and treatment time to compare vehicle and DEX treated cells.

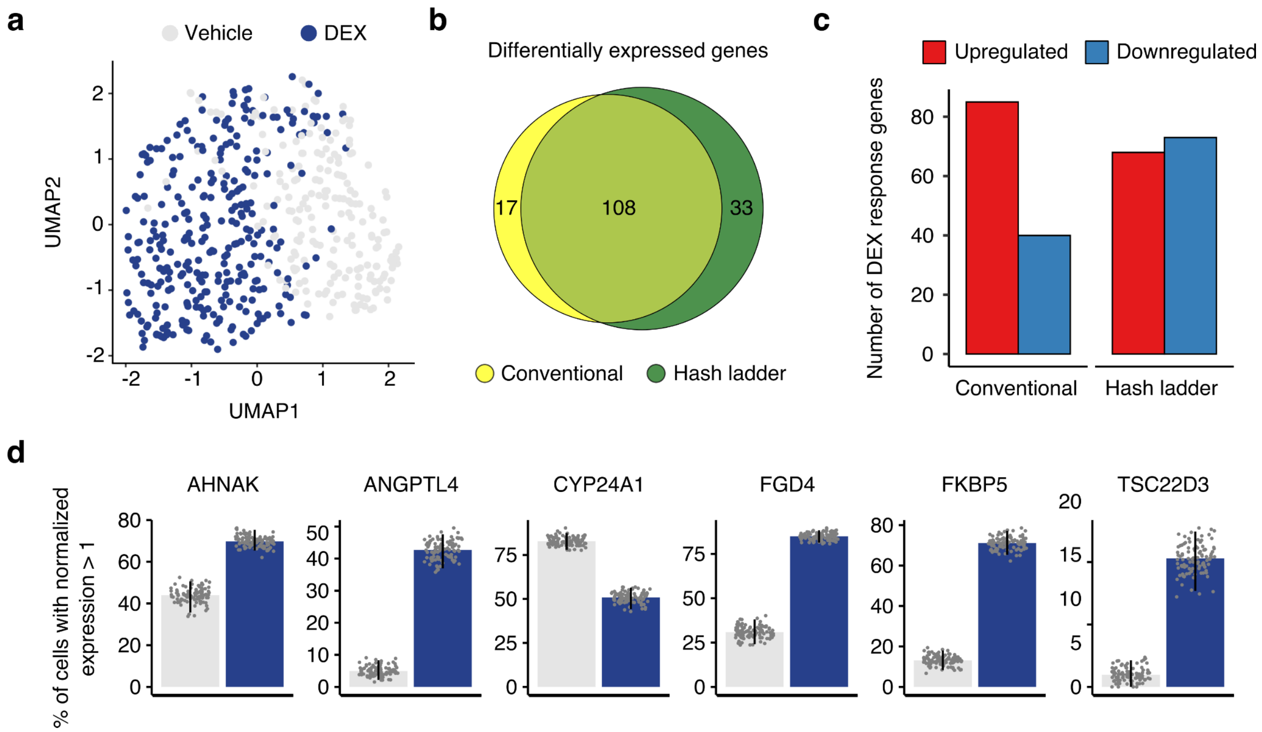

Supplementary Fig. 12: **Identification of DEX-induced transcriptional changes with hash ladder-based and conventional normalization.** (**a**) UMAP projections of vehicle and DEX treated A549 cells in the absence of HDAC inhibitors using the conventional size-factor normalized gene expression values. (**b**) Venn diagram showing the overlap of differentially expressed DEX response genes using the two normalization approaches. (**c**) Barplot showing number of upregulated and downregulated DEX response genes found using the conventional and hash ladder normalization approaches. (**d**) Hash ladder normalized expression levels of known DEX response genes. Bars represent the percentage of cells with normalized expression value greater than 1, and the error bars show the 95% confidence interval obtained using a bootstrap method (n = 100 bootstrap samples).

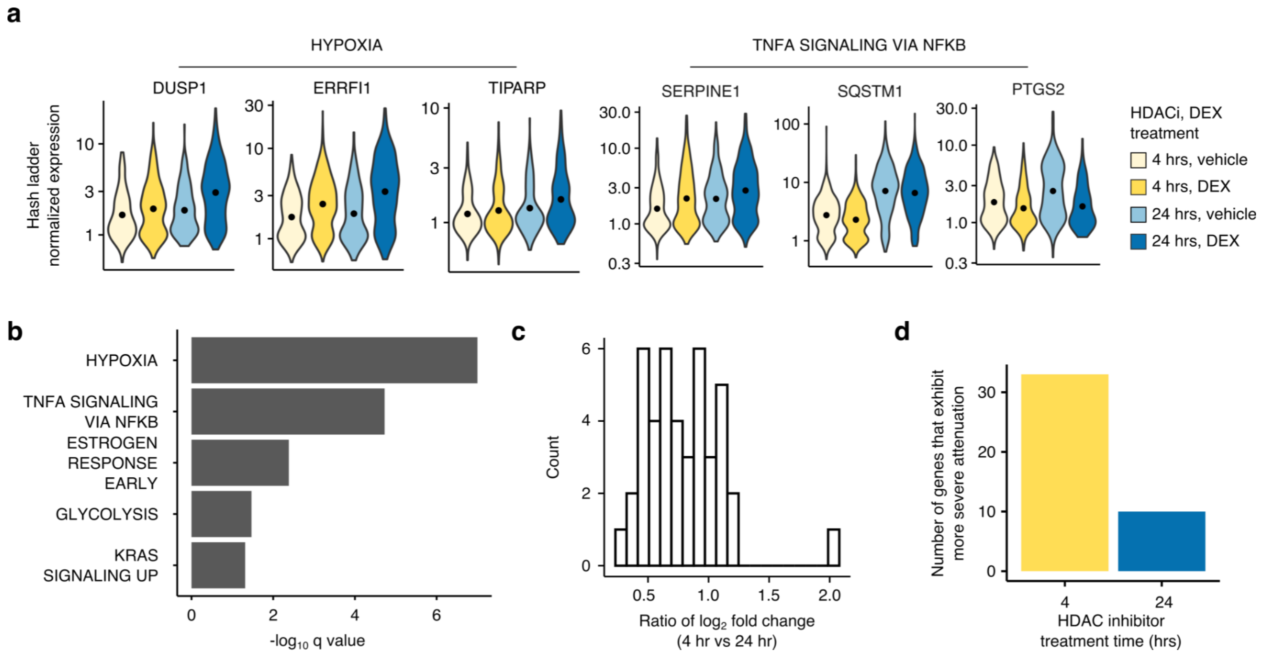

Supplementary Fig. 13: **HDAC inhibition attenuates DEX-induced transcriptional changes.** (**a**) Violin plots showing the hash ladder normalized expression values of genes that were induced by DEX in the presence of HDAC inhibitors. The expression values of genes involved in hypoxia response and TNF-A signaling are shown. (**b**) Barplot showing the classification of DEX response genes in the presence of HDAC inhibitors using the MSigDB hallmark gene sets (FDR < 0.05). (**c**) Histogram showing the ratio of log2 fold changes (DEX vs. vehicle) of average expression values of the shared DEX response genes between the 4 hour and 24 hour HDAC inhibitor treated cells. (**d**) Barplot summarizing whether the induction of DEX responsive genes are more severely impaired in 4 vs. 24 hour HDAC inhibitor treated cells using the log2 fold change ratio from Supplemental Fig. 10c.

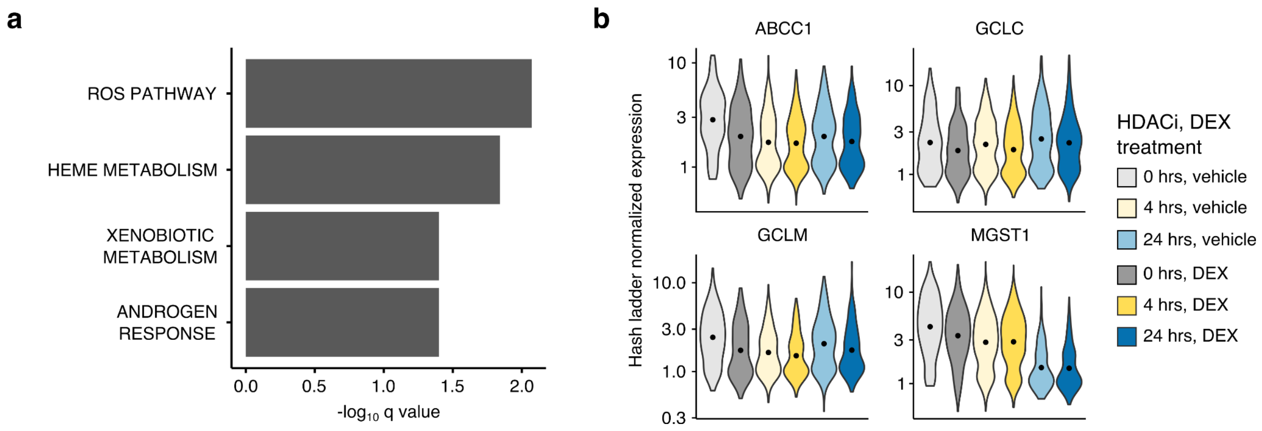

Supplementary Fig. 14: **HDAC inhibition blocks DEX-induced transcriptional changes.** (**a**) Barplot showing the classification of unresponsive DEX genes that are shared between the 4 and 24 hour HDAC inhibitor treatment conditions, using the MSigDB hallmark gene sets (FDR < 0.05). (**b**) Violin plots showing the hash ladder normalized expression values of unresponsive DEX genes that are involved in reactive oxygen species (ROS) pathway.

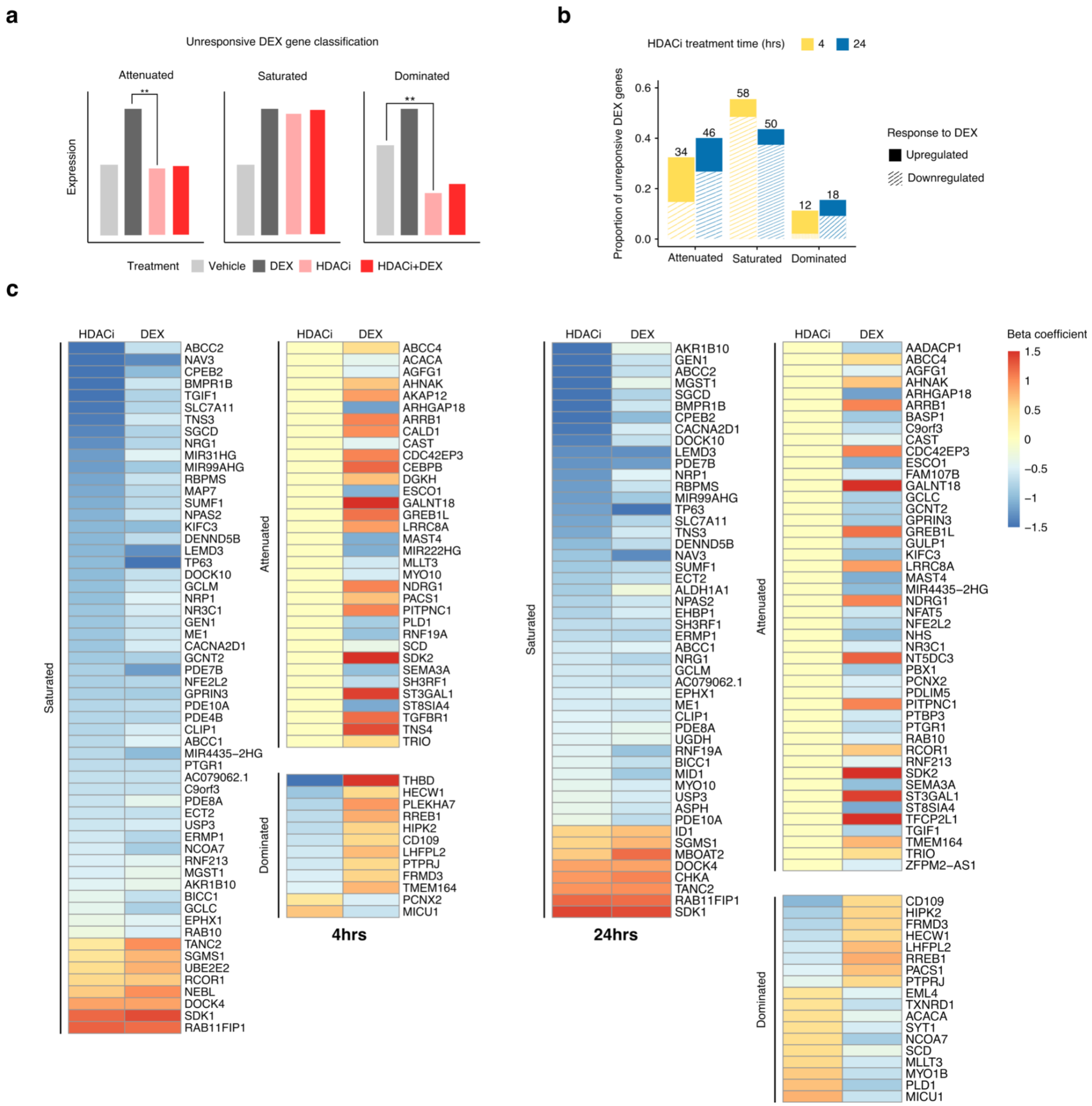

Supplementary Fig. 15: Classification of normally DEX-induced genes that failed to respond to DEX as a consequence of HDAC inhibition. (**a**) Illustration of gene expression patterns in each gene category. (**b**) Proportion of unresponsive DEX genes for each category, colored by HDAC inhibitor treatment time. (**c**) Heatmap of beta coefficient values of unresponsive DEX genes after 4 hours (left) and 24 hours (right) of HDACi treatment. Rows represent genes and columns represent beta coefficients (FDR < 0.01) for terms that capture effects from HDACi treatment alone and DEX treatment alone.

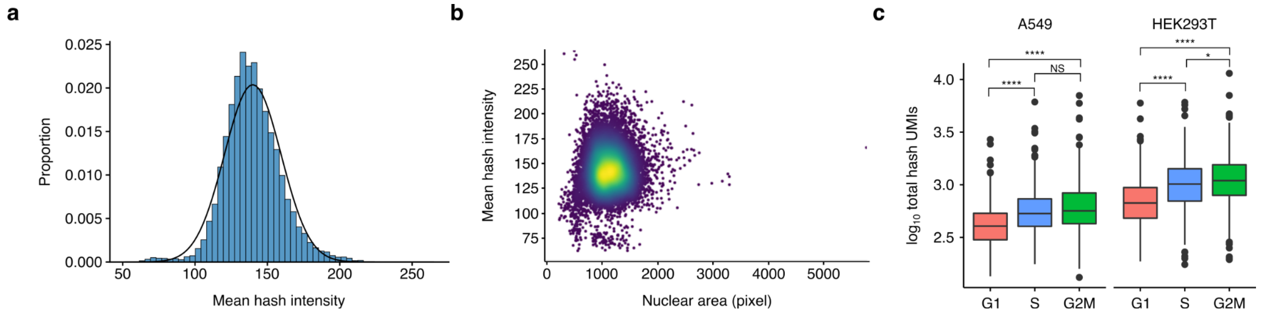

Supplementary Fig. 16: Hash oligo uptake is modestly influenced by cell size and cycle stages. (**a**) Histogram of mean hash oligo intensity values for A549s. These cells were labeled with fluorescently labeled hash oligos and assessed via microscopy. (**b**) Scatterplot showing the relationship between nuclear area and mean hash oligo intensity. (**c**) Boxplot of $\log_{10}$ hash UMIs for A549 and HEK293T in different cell cycle stages (n = 2378 cells). These cells were labeled with DAPI and each cell cycle group was sorted by FACS. The centerline of the boxplots indicates the median, the box displays the first and third quartile, and the whiskers show the 1.5 IQR. Outlier values are displayed as points. *P < 0.05, ****P<0.0001; two-tailed t-tests.

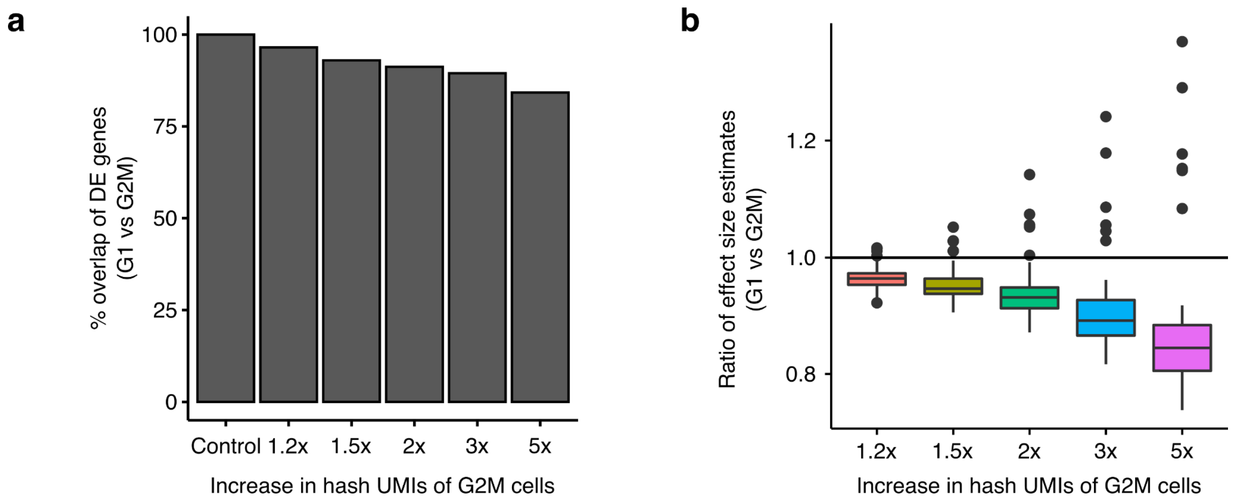

Supplementary Fig. 17: Artificial increase in hash UMIs in G2M cells does not significantly impact differential expression analysis. (**a**) Barplot showing the % of DE genes recovered after intentionally increasing total hash UMI counts of A549 cells in the G2M stage. (**b**) Boxplot comparing the ratio of effect size estimates of overlapping DE genes recovered from (**a**) (n = 48 genes). The centerline of the boxplots indicates the median, the box displays the first and third quartile, and the whiskers show the 1.5 IQR. Outlier values are displayed as points.

Supplementary Table 1. Differential expression analysis using flavopiridol pseudotime as covariate (Wald test; Benjamini-Hochberg adjusted $p < 0.01$), performed with the conventional and hash ladder-based normalized expression values.

Supplementary Table 2. Differential expression analysis using HDAC inhibitor pseudotime as covariate (Wald test; Benjamini-Hochberg adjusted $p < 0.01$), performed with the conventional and hash ladder-based normalized expression values.

Supplementary Table 3. Gene set enrichment analysis of highly differentially expressed genes along the HDACi pseudotime trajectory using the hash ladder-based normalization approach.

Supplementary Table 4. Differential expression analysis between vehicle and DEX treated cells in the absence of HDAC inhibitors (Wald test; Benjamini-Hochberg adjusted $p < 0.05$), performed with the conventional and hash ladder-based normalized expression values.

Supplementary Table 5. Differential expression analysis between vehicle and DEX treated cells in the presence of HDAC inhibitors (Wald test; Benjamini-Hochberg adjusted $p < 0.05$), performed with the conventional and hash ladder-based normalized expression values.

Supplementary Table 6. Hash oligo sequences and the amount of each hash species spiked in for each experiment.
