# Supplementary material for: Nuclear oligo hashing improves differential analysis of single-cell RNA-seq
Source: Nat Commun. 2022 May 13;13:2666. doi: 10.1038/s41467-022-30309-4 (PMC9106741; doi:10.1038/s41467-022-30309-4)
Supplement: Supplementary file 2 — Reporting Summary [file 41467_2022_30309_MOESM2_ESM.pdf]

nature research

Corresponding author(s): Cole Trapnell
Last updated by author(s): Mar 16, 2022

Reporting Summary

Nature Research wishes to improve the reproducibility of the work that we publish. This form provides structure for consistency and transparency in reporting. For further information on Nature Research policies, see our Editorial Policies and the Editorial Policy Checklist.

Statistics

For all statistical analyses, confirm that the following items are present in the figure legend, table legend, main text, or Methods section.

n/a	Confirmed	
☐	☒	The exact sample size (*n*) for each experimental group/condition, given as a discrete number and unit of measurement
☐	☒	A statement on whether measurements were taken from distinct samples or whether the same sample was measured repeatedly
☐	☒	The statistical test(s) used AND whether they are one- or two-sided<br>*Only common tests should be described solely by name; describe more complex techniques in the Methods section.*
☐	☒	A description of all covariates tested
☐	☒	A description of any assumptions or corrections, such as tests of normality and adjustment for multiple comparisons
☐	☒	A full description of the statistical parameters including central tendency (e.g. means) or other basic estimates (e.g. regression coefficient) AND variation (e.g. standard deviation) or associated estimates of uncertainty (e.g. confidence intervals)
☐	☒	For null hypothesis testing, the test statistic (e.g. *F*, *t*, *r*) with confidence intervals, effect sizes, degrees of freedom and *P* value noted<br>*Give P values as exact values whenever suitable.*
☒	☐	For Bayesian analysis, information on the choice of priors and Markov chain Monte Carlo settings
☒	☐	For hierarchical and complex designs, identification of the appropriate level for tests and full reporting of outcomes
☐	☒	Estimates of effect sizes (e.g. Cohen's *d*, Pearson's *r*), indicating how they were calculated

Our web collection on statistics for biologists contains articles on many of the points above.

Software and code

Policy information about availability of computer code

Data collection	No software was used to collect data in this study.
Data analysis	Previously published scripts (https://github.com/cole-trapnell-lab/sci-plex) and the single cell analysis package Monocle3 (version 0.2.0) were used in this study. A copy of the analyses performed is available on GitHub (https://github.com/khj3017/hash_ladder). Softwares used include bcl2fastq v2.20, cutadapt v1.18, trim_galore v0.6.5, STAR v2.6.1d, samtools v1.9, bedtools v2.27.1, datamash v1.3, and R v3.6.1.

For manuscripts utilizing custom algorithms or software that are central to the research but not yet described in published literature, software must be made available to editors and reviewers. We strongly encourage code deposition in a community repository (e.g. GitHub). See the Nature Research guidelines for submitting code & software for further information.

Data

Policy information about availability of data

All manuscripts must include a data availability statement. This statement should provide the following information, where applicable:

- Accession codes, unique identifiers, or web links for publicly available datasets
- A list of figures that have associated raw data
- A description of any restrictions on data availability

Raw and processed data can be downloaded on GEO under accession number GSE166470 (https://www.ncbi.nlm.nih.gov/geo/query/acc.cgi?acc=GSE166470) and available on our GitHub repository (https://github.com/khj3017/hash_ladder).

Field-specific reporting

Please select the one below that is the best fit for your research. If you are not sure, read the appropriate sections before making your selection.

- [x] Life sciences
- [ ] Behavioural & social sciences
- [ ] Ecological, evolutionary & environmental sciences

For a reference copy of the document with all sections, see nature.com/documents/nr-reporting-summary-flat.pdf

Life sciences study design

All studies must disclose on these points even when the disclosure is negative.

Sample size	No explicit calculations were performed to determine sample size. A typical sci-RNA-seq experiment can profile transcriptomes of 1000-7000 cells. We reasoned that profiling > 200 cells per condition in a single experiment should be sufficient to observe the necessary transcriptomic changes induced by the drugs.
Data exclusions	No data were excluded in this study.
Replication	The flavopiridol timecourse experiment (Figure 2) was performed with one replicate per condition but the results were validated using bulk RNA-seq with ERCC spike-ins. For HDAC inhibitor timecourse experiment (Figure 3), each treatment condition was performed in two replicate wells. For HDAC inhibitor and dexamethasone co-treatment experiment (Figure 4), each treatment condition was performed in two replicate wells.
Randomization	The order of samples were randomized during drug treatments and library preparation.
Blinding	Investigators were blinded to group allocation during data collection, sequencing, and analysis.

Reporting for specific materials, systems and methods

We require information from authors about some types of materials, experimental systems and methods used in many studies. Here, indicate whether each material, system or method listed is relevant to your study. If you are not sure if a list item applies to your research, read the appropriate section before selecting a response.

Materials & experimental systems

n/a	Involved in the study	
x		Antibodies
	x	Eukaryotic cell lines
x		Palaeontology and archaeology
	x	Animals and other organisms
x		Human research participants
x		Clinical data
x		Dual use research of concern

Methods

n/a	Involved in the study	
x		ChIP-seq
x		Flow cytometry
x		MRI-based neuroimaging

Eukaryotic cell lines

Policy information about cell lines

Cell line source(s)	HEK293T embryonic kidney cells (CRL-3216) and A549 lung epithelial cells (CCL-185) were purchased from ATCC.
Authentication	None of the cell lines were authenticated.
Mycoplasma contamination	HEK293T and A549 cells were tested and confirmed negative for mycoplasma contamination.
Commonly misidentified lines (See ICLAC register)	No commonly misidentified cell lines were used.

Animals and other organisms

Policy information about studies involving animals; ARRIVE guidelines recommended for reporting animal research

Laboratory animals	For Supplementary Fig. 2, we used female and male wildtype AB zebrafish raised at 28.5C under 14:10 light:dark cycles for 24 hpf.
Wild animals	The study did not involve wild animals.
Field-collected samples	The study did not involve samples collected from the field.

Ethics oversight	All procedures involving live animals followed federal, state and local guidelines for humane treatment and protocols approved by Institutional Animal Care and Use Committees of the University of Washington.

Note that full information on the approval of the study protocol must also be provided in the manuscript.
